# Association of peripheral inflammatory indicators with osteoarthritis risk

**DOI:** 10.1016/j.ocarto.2024.100496

**Published:** 2024-06-19

**Authors:** Shiyong Zhang, Yanlin Zhong, Xudong Wang, Wei Jiang, Xicong Chen, Yunze Kang, Zhiwen Li, Weiming Liao, Linli Zheng, Puyi Sheng, Ziji Zhang

**Affiliations:** a Department of Joint Surgery, The First Affiliated Hospital, Sun Yat-sen University, Guangzhou, Guangdong 510080, China; b Department of Bone and Joint, Shenzhen People's Hospital (The Second Clinical Medical College, Jinan University, The First Affiliated Hospital, Southern University of Science and Technology), Shenzhen 518020, Guangdong, China; c The Tenth Department of Orthopedics, Foshan Hospital of Traditional Chinese Medicine, China

**Keywords:** Osteoarthritis, Peripheral inflammation, UK biobank, Prospective cohort, Inflammatory indicators

## Abstract

**Objectives:**

Numerous studies have established the role of inflammation in osteoarthritis (OA) progression, yet limited research explores the association between systemic inflammatory indicators and pre-diagnosis OA risk. This study aimed to investigate the association between peripheral inflammatory indicators and the risk of OA using data from the UK Biobank.

**Methods:**

The study analyzed data from 417,507 participants in the UK Biobank, including neutrophil count, lymphocyte count, monocyte count, platelet count, and C-reactive protein meter. Additionally, derived ratios such as NLR(neutrophils-lymphocytes ratio), PLR(Platelets-lymphocytes ratio), SII(systemic immune-inflammation index), and LMR (lymphocytes-monocytes ratio) were examined. Cox proportional hazards models and restricted cubic spline models were used to assess both linear and nonlinear associations.

**Results:**

Over a mean follow-up period of 12.7 years, a total of 49,509 OA events were identified. The findings revealed that CRP (HR:1.06, 95%CI:1.05–1.07), NLR (HR:1.02, 95%CI:1.01–1.03), PLR (HR:1.02, 95%CI:1.01–1.03), and SII (HR:1.03, 95%CI:1.01–1.04) were associated with an increased risk of OA, while LMR (HR:0.97, 95%CI:0.96–0.99) showed a significant negative correlation with OA risk. Subgroup analyses further emphasized that these associations were significant across most of the population. Although neutrophils, lymphocytes, monocytes, and platelets showed a nominal association with the risk of OA, the results were unreliable, especially for specific joint OA.

**Conclusion:**

The study provides evidence of a significant association between elevated peripheral inflammatory indicators and OA risk. These findings underscore the importance of low-grade chronic inflammation in OA development. The potential clinical utility of these indicators as early predictors of OA is suggested, warranting further exploration.

## 1 Introduction

Osteoarthritis (OA) is a degenerative disease that is increasingly common and a major cause of pain, disability, and reduced quality of life in the elderly population [1]. While it was previously believed to be a wear-and-tear disease, new evidence suggests that the causes of OA are multidimensional [[2], [3], [4]]. Numerous studies have confirmed the significant role of inflammation in the occurrence and progression of OA [5]. However, due to the chronic and low-grade nature of inflammation in OA, it is challenging to assess the inflammatory status before diagnosing OA, and there is ongoing debate regarding the selection of inflammation indicators [[6], [7], [8]]. Therefore, it is crucial to identify stable peripheral inflammation indicators and explore their association with the occurrence of OA. This research will contribute to a better understanding of the mechanism of OA and aid in predicting the risk of developing OA.

Neutrophils and lymphocytes are crucial cells in both innate and adaptive immunity, playing an important role in regulating inflammation and diagnosing diseases [9,10]. The innate immune system is the first to exert regulatory functions on tissue injury or inflammation [11]. However, the counting of blood cells is limited by instrumentation and technology, resulting in a wide range of absolute values that cannot be directly compared. Recent evidence suggests that inflammatory indicators based on peripheral immune markers, such as NLR (neutrophils-lymphocytes ratio), PLR (Platelets-lymphocytes ratio), SII (systemic immune-inflammation index), and LMR (lymphocytes-monocytes ratio), hold important value in predicting various diseases [12,13]. Retrospective analysis of literature-based evidence reveals that NLR, PLR, and LMR, as indicators of whole blood inflammatory response, are associated with OA activity [14]. Some cross-sectional studies also suggest that novel indicators like NLR can predict the progression and severity of OA [15]. Therefore, reports indicate that the role of peripheral immune cells in the progression of OA has been significantly underestimated [16].

Current assessments of these associations remain incomplete and lack exploration of specific OA. In this study, we conducted a prospective analysis using data from the UK Biobank to evaluate the association between multiple peripheral inflammation indicators and OA, including both linear and nonlinear associations. We also assessed the potential of these indicators as predictors for the occurrence and progression of OA. The UK Biobank is a comprehensive cohort study consisting of more than 500,000 volunteers, the majority of whom provided blood samples at the beginning of the study [17].

## 2 Materials and methods

### 2.1 Participants and data access

**Fig. 1:**
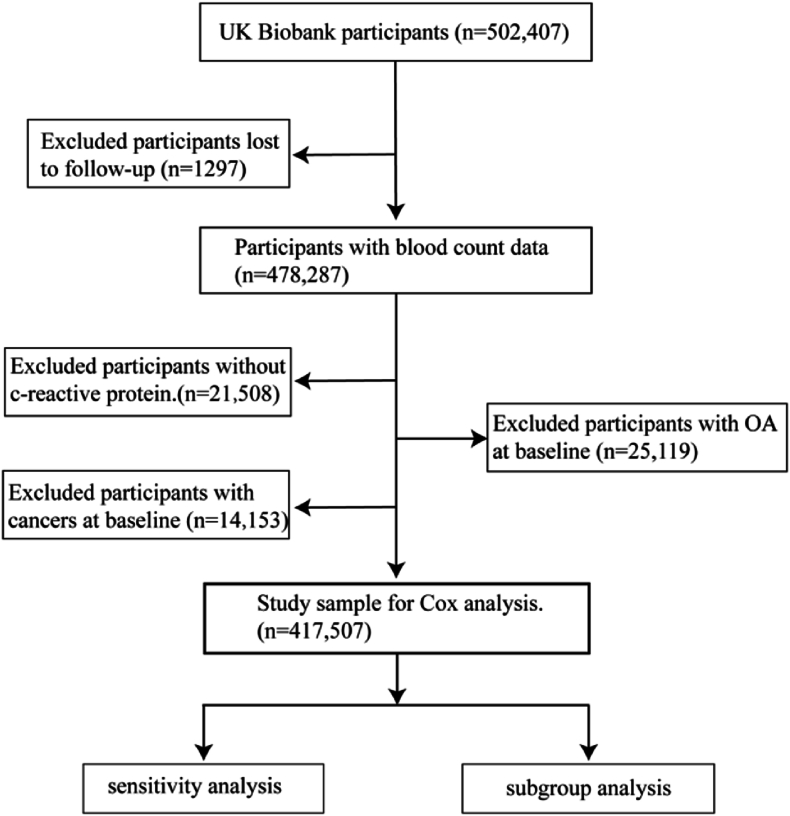
Flow diagram of study design.

We used the UK Biobank database as the data source (application number 71986). All participants in the UK Biobank provided informed written consent at the time of inclusion in the cohort, and all information was available for scientific research. When selecting study subjects, we initially excluded participants who were lost to follow-up (n ​= ​1297). Then, participants with available blood data were included. Subsequently, we excluded participants with incomplete peripheral markers (n ​= ​21,508), participants who had been diagnosed with OA before baseline (n ​= ​25,199), and those reported any history of cancer (n ​= ​14,153). The detailed screening process is illustrated in the flow chart (Fig. 1).

### 2.2 Peripheral inflammatory indicators

The UK Biobank conducted quality control and reporting on blood collection, testing, and data compilation. Detailed methods and information on the instruments used are publicly available biobank.ndph.ox.ac.uk/ukb/ukb/docs/haematology.pdf. We specifically chose five peripheral whole blood markers: neutrophil count, lymphocyte count, monocyte count, platelet count, and C-reactive protein count. Additionally, we calculated the ratios of neutrophils to lymphocytes (NLR), platelets to lymphocytes (PLR), systemic immune-inflammation index (SII, neutrophils ​× ​platelets/lymphocytes), and lymphocytes to monocytes (LMR) for each participant. Previous research has demonstrated that these ratios serve as reliable indicators of the body's inflammatory status [12,14]. Some participants may have had multiple indicators in the database, and we primarily used blood test data at baseline.

### 2.3 Outcome ascertainment

Information on disease diagnoses in the UK Biobank database was categorized by professionals using ICD-10 codes and structured spreadsheets. We queried the database according to the ICD-10 codes for OA events registered in 2006–2022 and identified most OA events. We excluded inflammatory joint disease, infectious joint disease, and post-traumatic joint OA [18] (supplementary table 1). The diagnostic information primarily comes from primary care, hospital admission data, and self-report. Some participants have multiple instances of diagnostic information, but we used the first diagnosis as the outcome event. Participants were followed from initial recruitment until the first diagnosis of OA, death, loss to follow-up, or the end (December 31, 2022).

### 2.4 Covariates

Sociodemographic information and lifestyle factors, such as age, gender, Index of Multiple Deprivation (IMD), education, and income level, were collected using a touchscreen questionnaire [19]. Smoking status was categorized as current, past, or never. Drinking frequency was assessed as daily or almost daily, 1–4 times per week, 1–3 times per month, or occasional/never. Exercise was measured using the total metabolic equivalent of task (MET) in a week. Vegetable and fruit intake was recorded as ≥5 servings per day (yes or no). Mineral and vitamin intake was also recorded (yes or no). Obesity is defined by body mass index (BMI), which is calculated as weight (kg) divided by the square of height (m). According to the World Health Organization definition, a BMI ≥29.9 ​kg/m^2^ is considered obesity. Income is categorized into low, medium, and high tiers based on total pre-tax household income. Participants selected income options in the questionnaire that matched their income: < £18,000, £18,000-£30,999, £31,000-£51,999, £52,000-£100,000, > £100,000, do not know, or prefer not to answer. Education was divided into two levels: high (college or university degree, other professional qualifications) and low [20].

### 2.5 Statistical analysis

Results for continuous variables were reported as mean and standard deviation (SD), while categorical variables were expressed as number and percentage. To compare effect sizes between different indicators, peripheral inflammation indicators were log-transformed and standardized to Z-scores (Z = (value - mean)/SD). Therefore, the hazard ratio (HR) represents the associated effect per SD increase in peripheral inflammation indicators. Cox proportional hazards regression models were used to assess potential associations between various peripheral inflammation indicators and the risk of OA. Model 1 adjusted only for age and gender. Model 2 adjusted for gender, age, obesity, education, and income. Model 3 added adjustments for lifestyle factors (smoking, research, exercise, vegetable & fruit intake, mineral and vitamin intake). Additionally, restricted cubic spline (RCS) analysis was conducted to explore the potential nonlinear relationship between peripheral inflammation indicators and OA risk, using a three-part model at the 10th, 50th, and 90th percentiles to flexibly model these associations. The proportional risk hypothesis was tested using the Schoenfeld residual method, and no violations were observed.

To ensure the reliability of our findings, we conducted several additional analyses. Firstly, we repeated the analysis by excluding all extreme exposure values (greater than ±3 standard deviations). Secondly, to reduce the effects of selection bias and covariates, we used propensity score matching. We weighted each confounding factor and then proximity matched with a variable ratio of one-to-many (1:2) within the caliper. Thirdly, we excluded participants with less than 2 and 5 years of follow-up, respectively, and performed the analysis again. Finally, we analyzed the effect of CRP and four peripheral inflammatory indicators on the long-term cumulative risk of OA using Kaplan-Meier curves, and the group median was used as the cut-off value between the high and low groups. Additionally, we performed several subgroup analyses including gender (male, female), age (<60 years, ≥60 years), obesity (obese, normal), smoking status (current/precious, never), alcohol consumption (drinking, occasional/never), activity level (MET< 600, MET ≥600), income (<£30,999, ≥£30,999), and education level. To explore the association of peripheral inflammatory indicators with OA risk in different populations. All analyses were performed using R software (Windows, version 4.2.2). Statistical tests were two-sided, and *p* values less than 0.05 were considered statistically significant differences.

## 3 Results

A total of 417,507 participants (males: 171,916, females: 196,082) were included in this study. The participants had an average age of 56.25 years (SD ​= ​8.13). Over a mean follow-up period of 12.7 years, a total of 49,509 OA events were identified. In comparison to non-OA participants (Table 1), OA patients were more likely to be female, have lower-middle income, lower educational background, higher deprivation index, and higher rates of obesity. At baseline, participants in the OA group had slightly higher peripheral immune markers and inflammatory indicators than those in the control group.

**Table 1:** Baseline characteristics of the UK biobank cohort stratified by incident osteoarthritis status.

Characteristics	Incident Osteoarthritis		Overall n ​= ​417,507
	No (n ​= ​367,998)	Yes (n ​= ​49,509)	
Mean (SD) age, years	56.25 (8.13)	59.95 (7.09)	56.69 (8.10)
**Gender**			
Male, N (%)	171,916 (46.7)	20,783 (42.0)	192,699 (46.2)
Female, N (%)	196,082 (53.3)	28,726 (58.0)	224,808 (53.8)
**Ethnic, (white) N (%)**	347,609 (94.5)	47,529 (96.0)	395,138 (94.6)
**Income, N (%)**			
High	88,157 (24.0)	7066 (14.3)	95,223 (22.8)
Medium	162,691 (44.2)	21,906 (44.2)	184,597 (44.2)
Low	117,150 (31.8)	20,537 (41.5)	137,687 (33.0)
Mean (SD) IMD	17.07 (13.91)	17.98 (14.43)	17.17 (13.98)
Obesity, N (%)	123,523 (33.6)	24,568 (49.7)	148,091 (35.5)
**Education, N (%)**			
High	143,385 (39.0)	15,138 (30.6)	158,523 (38.0)
Low	224,613 (61.0)	13,138 (69.4)	258,984 (62.0)
Fruit & vegetable, N (%)	136,615 (37.1)	20,264 (40.9)	156,879 (37.6)
Vitamin, N (%)	54,161 (14.7)	8272 (16.7)	62,433 (15.0)
Mineral, N (%)	78,650 (21.4)	11,172 (22.6)	89,822 (21.5)
**Alcohol consumption, N (%)**			
Daily or almost daily	75,370 (20.5)	9925 (20.0)	85,295 (20.4)
1–4 times a week	182,673 (49.6)	23,493 (47.5)	206,166 (49.4)
1–3 times a month	40,998 (11.1)	5398 (10.9)	46,396 (11.1)
Special occasions only/Never	68,957 (18.7)	10,693 (21.6)	79,650 (19.1)
Median (IQR) Physical activity, MET hours/week	23.95 (31.22)	25.59 (33.25)	24.15 (31.47)
**Smoking status(%)**			
Current	39,616 (10.8)	4666 (9.4)	44,282 (10.6)
Previous	122,598 (33.3)	19,565 (39.5)	142,163 (34.1)
Never	205,784 (55.9)	25,278 (51.1)	231,062 (55.3)
Neutrophil count (mean (SD))	4.21 (1.40)	4.29 (1.42)	4.22 (1.40)
Monocyte count (mean (SD))	0.47 (0.21)	0.48 (0.21)	0.47 (0.21)
Lymphocyte count (mean (SD))	1.96 (0.87)	1.98 (0.71)	1.96 (0.85)
Platelet count (mean (SD))	252.95 (59.51)	255.25 (59.82)	253.23 (59.55)
CRP (mean (SD))	2.44 (4.14)	3.09 (4.69)	2.52 (4.21)
NLR (mean (SD))	2.34 (1.21)	2.36 (1.28)	2.34 (1.22)
SII (mean (SD))	594.27 (354.66)	605.08 (380.63)	595.55 (357.85)
PLR (mean (SD))	141.42 (60.41)	140.98 (63.24)	141.36 (60.75)
LMR (mean (SD))	4.63 (3.65)	4.58 (2.77)	4.63 (3.56)

Data presented as mean (SD) for continuous variables and number (%) for categorical variables. Education level was categorized as high (college/university degree or other professional qualification) or low. The counting unit for neutrophils, monocytes, lymphocytes, and platelets is 10ˆ9 ​cells/Liter.

Abbreviations: CRP, C-reactive protein; NLR, neutrophils-lymphocytes ratio; LMR, lymphocytes-monocytes ratio; PLR, platelets-lymphocytes ratio; SII, systemic immune-inflammation index (neutrophils ​× ​platelets/lymphocytes).

### 3.1 Association of peripheral inflammation indicators with OA risk

Neutrophils and monocytes, representing innate immune cells, showed a statistically significant but not robust association with OA risk. Platelets, on the other hand, exhibited a statistically significant association with OA risk in the primary analysis. Each standard deviation (SD) increase in platelet count was associated with a 3% increased risk of OA, although this association was not observed in the sensitivity analysis. In contrast, adaptive immune lymphocytes demonstrated an inverse correlation with OA risk. Among the inflammatory indicators, CRP showed the most significant association with OA risk. For every SD increase in CRP, the risk of OA increased by 6%, and this association remained robust in all models (Table 2).

**Table 2:** Risk of osteoarthritis according to peripheral inflammatory indicators in the UK Biobank.

	Hazard Ratio (95% CI)		
	Model 1 *p*	Model 2 *p*	Model 3 *p*
Neutrophils	1.06 (1.05, 1.07) <0.01	1.01 (1.00, 1.02) 0.07	1.01 (1.01, 1.02) <0.01
lymphocytes	1.02 (1.01, 1.03) 0.01	0.97 (0.95, 0.98) <0.01	0.97 (0.96, 0.99) <0.01
Monocytes	1.03 (1.03, 1.04) <0.01	1.00 (0.99, 1.02) 0.55	1.00 (0.99, 1.02) 0.43
Platelets	1.04 (1.03, 1.05) <0.01	1.03 (1.02, 1.04) <0.01	1.03 (1.02, 1.04) <0.01
C-reactive protein	1.10 (1.09, 1.11) <0.01	1.06 (1.05, 1.06) <0.01	1.06 (1.05, 1.07) <0.01
NLR	1.02 (1.01, 1.03) <0.01	1.02 (1.01, 1.02) <0.01	1.02 (1.01, 1.03) <0.01
PLR	1.00 (0.99, 1.01) 0.49	1.02 (1.01, 1.03) <0.01	1.02 (1.01, 1.03) <0.01
SII	1.03 (1.02, 1.04) <0.01	1.02 (1.02, 1.03) <0.01	1.03 (1.01, 1.04) <0.01
LMR	0.98 (0.96, 0.99) <0.01	0.97 (0.96, 0.98) <0.01	0.97 (0.96, 0.99) <0.01

Model 1: Adjusted for age and sex.

Model 2: Adjusted for age, sex, Index of Multiple Deprivation (IMD), obesity, income, and education.

Model 3: Adjusted for age, sex, Index of Multiple Deprivation (IMD), obesity, income, education, smoking, alcohol consumption, vitamin and mineral intake, and fruit & vegetable intake.

All exposures were log-transformed and standardized to z-scores, where the Hazard Ratio (HR) represents the predicted effect per standard deviation increment of the peripheral inflammatory indicators.

Additionally, novel inflammation indicators all exhibited robust associations with OA risk. Specifically, NLR(HR:1.02, 95%CI:1.01–1.03), PLR(HR:1.02, 95%CI:1.01–1.03), and SII(HR:1.03, 95%CI:1.01–1.04) were associated with an increased risk of OA, while LMR (HR:0.97, 95%CI:0.96–0.99) showed a significant negative correlation with OA risk (Table 2). Importantly, these associations were consistent across all models, suggesting minimal influence from confounding factors.

**Fig. 2:**
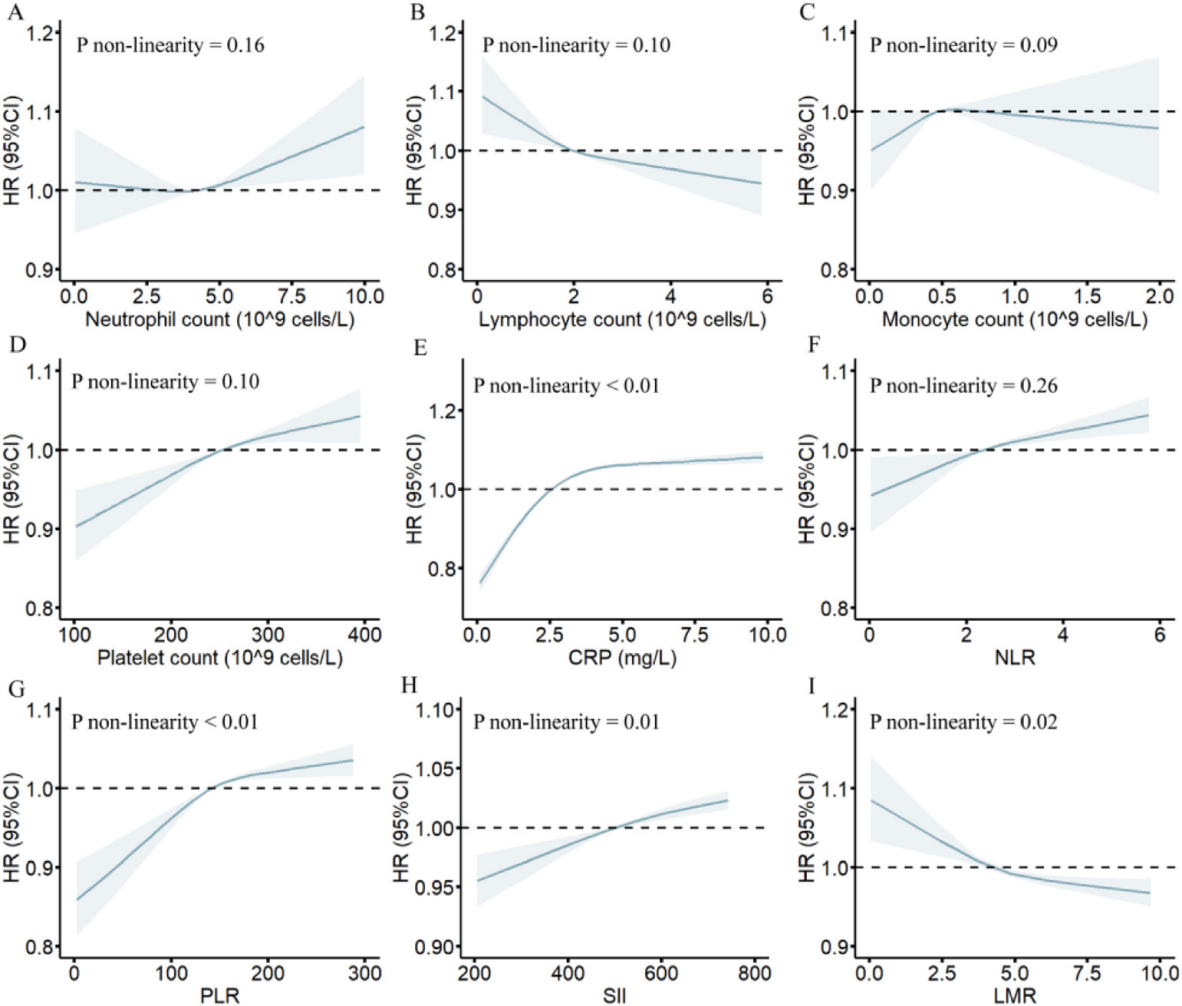
Estimated Non-linear Association Between Peripheral Inflammatory Indicators and the Risk of Osteoarthritis. Restricted cubic spline models were fitted for Cox proportional hazards models, and results were adjusted for age at baseline, sex, Index of Multiple Deprivation (IMD), obesity, income, education, smoking, alcohol consumption, vitamin and mineral intake, and fruit & vegetable intake. Subplots (A–I) represent the non-linear associations for each peripheral inflammatory indicator: (A) Neutrophils, (B) Lymphocytes, (C) Monocytes, (D) Platelets, (E) CRP (C-reactive protein), (F) NLR (neutrophils-lymphocytes ratio), (G) PLR (Platelets-lymphocytes ratio), (H) SII (systemic immune-inflammation index), (I) LMR (lymphocytes-monocytes ratio).

In a restricted cubic spline (RCS) further exploring the association between peripheral inflammatory markers and OA risk, we found no non-linear association between NLR (non-linear *p* ​= ​0.26) and all four immune markers with OA risk. Whereas CRP (nonlinear *p* ​< ​0.01), PLR (nonlinear *p* ​< ​0.01) and SII (nonlinear *p* ​= ​0.01) showed nonlinear associations with increased OA risk, LMR (nonlinear *p* ​= ​0.02) showed nonlinear associations with decreased OA risk (Fig. 2).

### 3.2 Subgroup and sensitivity analysis

**Fig. 3:**
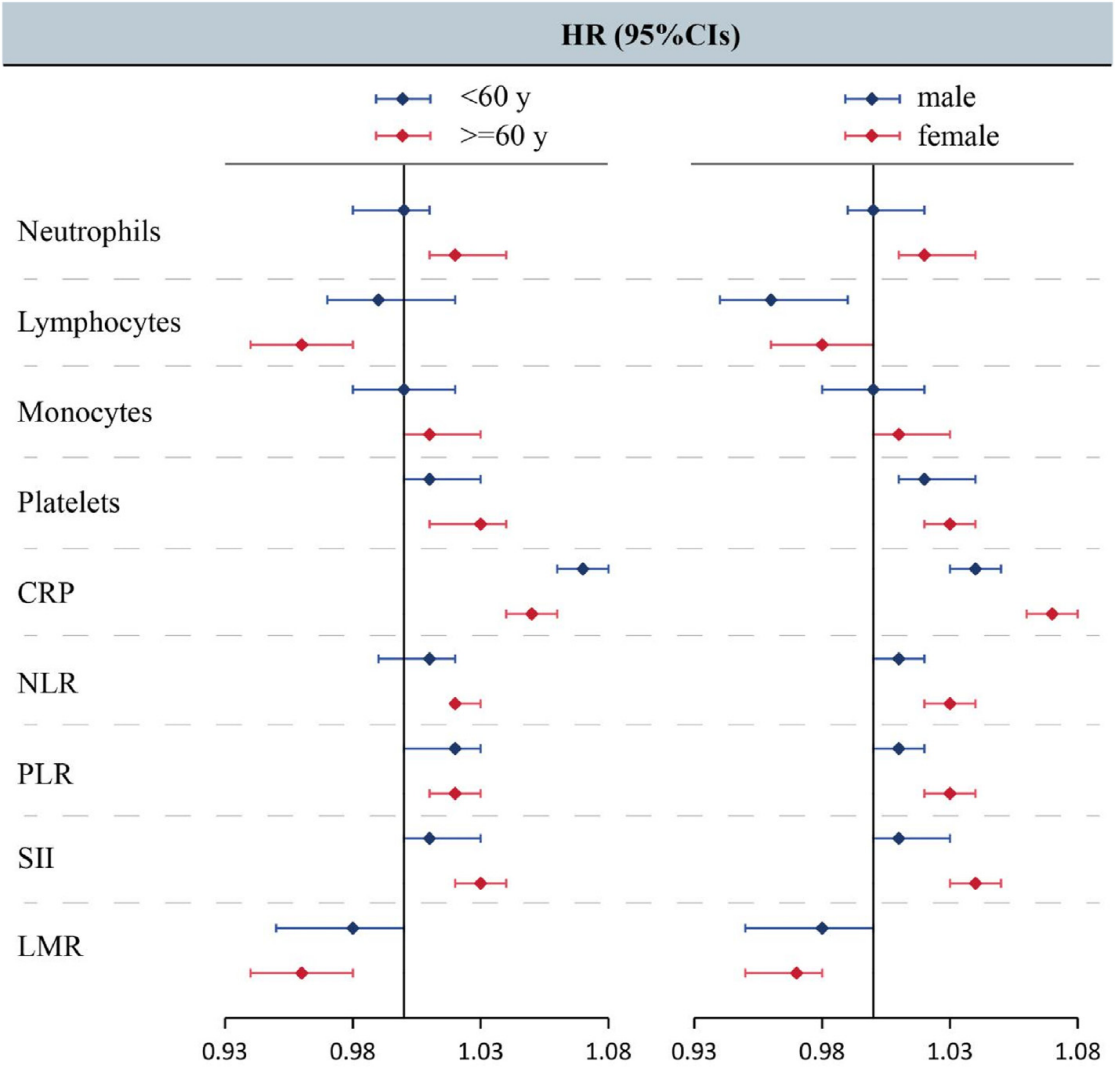
Association of Peripheral Inflammation Indicators with Osteoarthritis (OA) Risk in Subgroups. Models were adjusted for age, sex, Index of Multiple Deprivation (IMD), obesity, income, education, smoking, alcohol consumption, vitamin and mineral intake, and fruit & vegetable intake. All exposures were log-transformed and standardized to z-scores, where the Hazard Ratio (HR) represents the predicted effect per standard deviation increment of the peripheral inflammatory indicators.

Although we observed similar trends across subgroups as in the main analysis, the most notable differences were found within the sex and age subgroups. Among females, all inflammatory markers except monocytes were significantly associated with OA risk, whereas in males, including neutrophils, NLR, and LMR, the associations with OA risk were no longer statistically significant. In the older population, all inflammatory markers exhibited a significant association with OA risk. However, in the younger population, including all immune cells, platelets, NLR, and SII, there was no longer an association with OA risk (Fig. 3). Furthermore, within other subgroups of the population, including smoking, alcohol consumption, activity level, obesity, income, and education, the differences were mainly centered on monocytes and neutrophils, with all other inflammatory indicators showing consistent associations with OA risk (supplementary table 2).

Notably, significant differences were observed in the associations between peripheral inflammatory indicators and the risk of specific joint OA. Specifically, the associations between immune cell and platelet counts and specific joint OA risk became less robust. Despite this, novel inflammatory indicators continued to show stronger associations with OA risk across different joints, suggesting that these novel indicators may offer greater sensitivity and stability in reflecting systemic inflammation. Among the specific joint OA risks, the most significant associations were found between the novel inflammatory indicators and hip OA risk, followed by hand OA risk. The associations with knee OA risk, although statistically significant, were comparatively weaker (supplementary table 3).

**Fig. 4:**
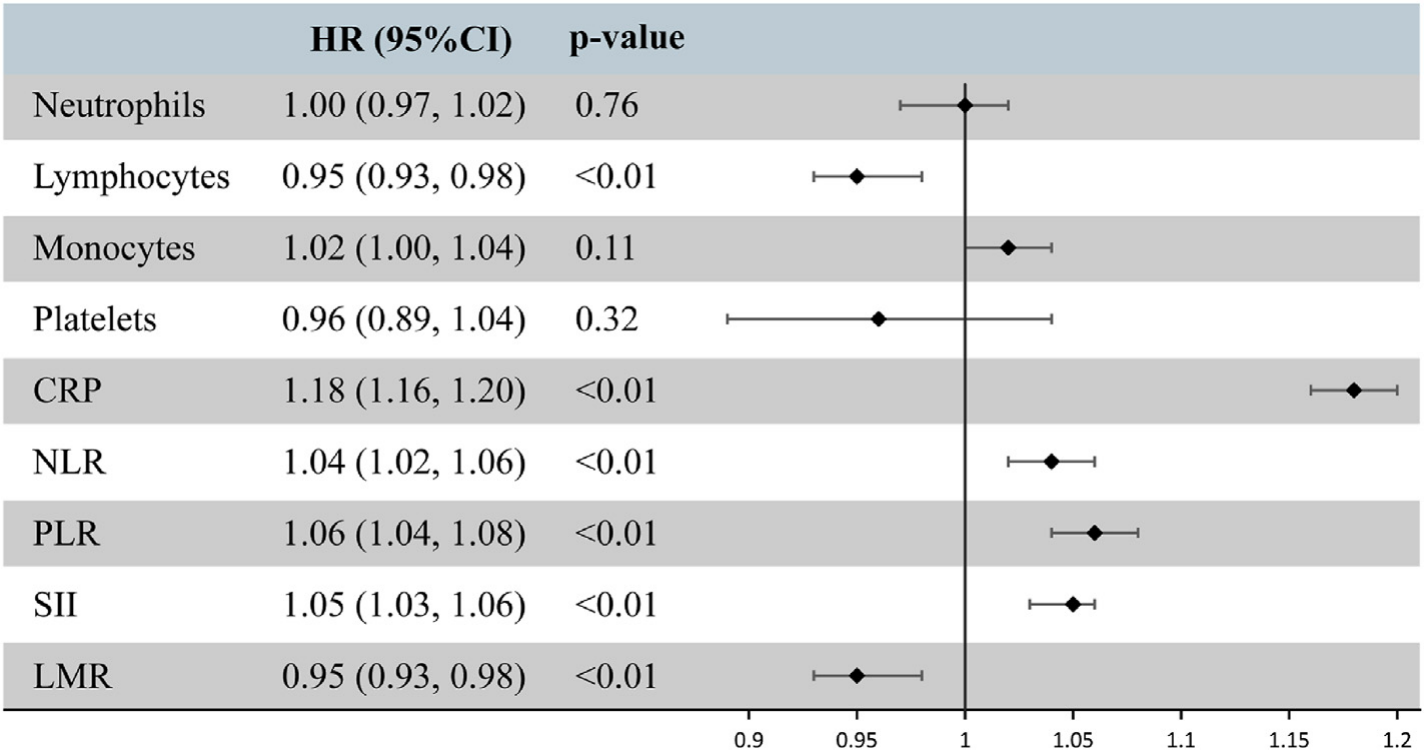
Association Analysis of Peripheral Inflammatory Indicators with Osteoarthritis (OA) Risk with Extreme Values Removed. Models were adjusted for age, sex, Index of Multiple Deprivation (IMD), obesity, income, education, smoking, alcohol consumption, vitamin and mineral intake, and fruit & vegetable intake. All exposures were log-transformed and standardized to z-scores, where the Hazard Ratio (HR) represents the predicted effect per standard deviation increment of the peripheral inflammatory indicators.

For the sensitivity analysis, we initially excluded participants with extreme exposure values. The results indicated that most of the associations between peripheral immune markers and the risk of OA lost statistical significance. In contrast, CRP (HR:1.18, 95%CI:1.16–1.20), NLR (HR:1.04, 95%CI:1.02–1.06), PLR (HR:1.06, 95%CI:1.04–1.08), SII (HR:1.05, 95%CI:1.03–1.06), along with LMR (HR:0.95, 95%CI:0.93–0.98), exhibited a more significant association with the risk of OA (Fig. 4). Interestingly, this association was further strengthened after propensity score matching, especially for CRP (HR:1.31, 95%CI:1.28–1.33), Neutrophils (HR:1.10, 95%CI:1.08–1.12), Platelets (HR:1.10, 95%CI:1.07–1.14), and SII (HR:1.07, 95%CI:1.05–1.09). (supplementary table 4). Additionally, when we excluded participants with less than 2 and 5 years of follow-up, respectively, we obtained two highly robust results (supplementary table 5). Finally, the Kaplan-Meier curves exhibited trends that aligned with the primary analysis. Notably, there was a significant difference in the cumulative risk of long-term OA when comparing high and low inflammatory markers using population median cutoffs. All subgroup log-rank tests had p-values less than 0.001 (supplementary figure 1).

## 4 Discussion

In the present study, we discovered that baseline inflammatory indicators in peripheral whole blood were linked to the risk of OA. Although blood immune markers did not show a significant association with OA risk, the derived inflammatory indicators based on these markers demonstrated a significant association. Specifically, NLR, PLR, SII, and the conventional inflammatory indicator CRP were associated with an increased risk of OA, while LMR was negatively associated with OA risk. Subgroup analyses revealed significant correlations in all subgroups except for the young and male groups, where the associations were slightly unstable.

In recent years, the notion that chronic low-grade inflammation plays a role in the development and progression of OA has gained widespread acceptance [8,21]. Consequently, numerous studies have been conducted to identify suitable inflammatory indicators that can explain and predict the occurrence of OA. These include novel indicators such as NLR, PLR, SII, and LMR, as well as traditional indicators like CRP, which is a well-established protein for detecting inflammatory states in the body [6,22]. However, there is controversy surrounding the analysis of the association between CRP and OA risk [[23], [24], [25], [26]]. Our findings support the notion that there is an association between CRP and the risk of OA. Several cross-sectional studies have pointed out that the level of NLR is strongly associated with knee OA (KOA) severity and has the potential to predict KOA progression [15,27]. Moreover, In a separate prospective study，the researcher found that NLR normalized faster than CRP and was a better indicator of inflammation and infection in predicting inflammation development after arthroplasty [28]. Our findings do not support this trend. Although NLR was associated with an elevated risk of overall OA, there was no statistically significant correlation with the risk of KOA. Possible mechanistic explanations include the chronic and low-grade nature of inflammation in OA, which is primarily mediated by the innate immune system [7,29,30]. In this view, the aggregation of neutrophils around the joints and the destruction of articular cartilage by neutrophil elastase (NE) are considered indispensable for the progression of OA [31,32]. A recent Mendelian randomization (MR) analysis also revealed a causal association between adaptive immune T cells and a reduced risk of hip OA [33]. Similarly, our study found that adaptively immunized lymphocytes and LMR were associated with a lower risk of OA.

In addition, the association between two inflammatory indicators, PLR and SII, and the risk of OA has not been previously reported. However, these indicators have been shown to be significantly useful in detecting and predicting other inflammation-related diseases. A cross-sectional study conducted on 22,290 individuals found that SII is a superior systemic inflammatory warning marker in hypertensive patients [34]. Prospective studies, conducted on the UK Biobank cohort, have also confirmed that SII and PLR are associated with an increased risk of several cancers and dementia [13,35]. In our present study, we initially report that PLR and SII are consistently and significantly associated with an increased risk of OA. However, further studies may be necessary to support our findings. Overall, our study revealed several associations between indicators of peripheral inflammation and the risk of OA. However, it remains uncertain whether these markers reflect the severity of OA. We hypothesize that systemic inflammation, as indicated by these markers, plays a role in OA development. Investigating the trajectory of systemic inflammatory response following joint arthroplasty is an area that requires further exploration in future research.

Our study possesses several noteworthy strengths. Firstly, we have utilized extensive samples and long-term follow-up data from UK Biobank, enabling us to thoroughly investigate the association between exposure and outcome. Secondly, we have employed a prospective cohort study design, which offers advantages over cross-sectional studies. Finally, we employed multiple methods, including propensity score matching, to minimize the effects of confounding variables. Nonetheless, it is important to acknowledge certain limitations of our study: 1) Possible selection bias of “healthy volunteers” in the UK Biobank, which would limit the generalization of our findings. However, other comparable studies have suggested that the large size and heterogeneity of UK Biobank exposure measures provide valid scientific inferences of the association between exposure and outcome. 2) Due to the nature of observational study design, causal associations are difficult to determine and only suggest associations. 3) This study defined OA cases based on ICD-10 codes. The raw information was primarily sourced from primary care records, hospital admission data, and self-reports. Consequently, some cases may have been identified based solely on symptoms. Additionally, most individuals with OA seek medical help due to joint pain or functional problems, which do not necessarily coincide with the initial onset of the disease. However, chronic diseases, including OA, present challenges in pinpointing the exact time of initial onset and in providing timely diagnoses. 4) our study population primarily consisted of individuals from Europeans, which may restrict the generalizability of our findings.

## 5 Conclusion

The study found a significant correlation between elevated peripheral inflammatory indicators (CRP, NLR, PLR, SII, and LMR) and the risk of OA, providing further evidence of the crucial role inflammation plays in the development and progression of OA. These findings also indicate that peripheral inflammation indicators have promising clinical potential as early predictors of OA. However, additional research is required to explore this association and understand the underlying mechanisms.

## Ethics statement

This study was approved by the North West Multi-center Research Ethics Committee, the England and Wales Patient Information Advisory Group, and the Scottish Community Health Index Advisory Group (application number 71986). All participants provided written informed consent prior to data collection.

## Funding

This study was supported by the 10.13039/100014717National Natural Science Foundation of China (82172406, 82201719), the 10.13039/501100003453Natural Science Foundation of Guangdong Province (no. 2022A1515012279), and the Project funded by China Postdoctoral Science Foundation (No. 2023M744059).

## Data accessibility

UK Biobank is an open-access resource, and the study website https://www.ukbiobank.ac.uk/ has information on available data and access procedures.

Data sets used for the analysis will be made available under reasonable requests.

## Consent for publication

Not applicable.

## Declaration of competing interest

The authors declared that no competing interests exist.

## References

[1] Hunter D.J., Bierma-Zeinstra S. (2019). Osteoarthritis. Lancet.

[2] Yao Q., Wu X., Tao C., Gong W., Chen M., Qu M. (2023). Osteoarthritis: pathogenic signaling pathways and therapeutic targets. Signal Transduct. Targeted Ther..

[3] Zhang S., Wang L., Kang Y., Wu J., Zhang Z. (2023). Nanomaterial-based reactive oxygen species scavengers for osteoarthritis therapy. Acta Biomater..

[4] Coryell P.R., Diekman B.O., Loeser R.F. (2021). Mechanisms and therapeutic implications of cellular senescence in osteoarthritis. Nat. Rev. Rheumatol..

[5] Motta F., Barone E., Sica A., Selmi C. (2023). Inflammaging and osteoarthritis. Clin. Rev. Allergy Immunol..

[6] Vlad S.C., Neogi T., Aliabadi P., Fontes J.D., Felson D.T. (2011). No association between markers of inflammation and osteoarthritis of the hands and knees. J. Rheumatol..

[7] Robinson W.H., Lepus C.M., Wang Q., Raghu H., Mao R., Lindstrom T.M. (2016). Low-grade inflammation as a key mediator of the pathogenesis of osteoarthritis. Nat. Rev. Rheumatol..

[8] Huang Z.Y., Perry E., Huebner J.L., Katz B., Li Y.J., Kraus V.B. (2018). Biomarkers of inflammation - LBP and TLR- predict progression of knee osteoarthritis in the DOXY clinical trial. Osteoarthritis Cartilage.

[9] Uderhardt S., Martins A.J., Tsang J.S., Lämmermann T., Germain R.N. (2019). Resident macrophages cloak tissue microlesions to prevent neutrophil-driven inflammatory damage. Cell.

[10] Liew P.X., Kubes P. (2019). The neutrophil's role during health and disease. Physiol. Rev..

[11] Girbl T., Lenn T., Perez L., Rolas L., Barkaway A., Thiriot A. (2018). Distinct compartmentalization of the chemokines CXCL1 and CXCL2 and the atypical receptor ACKR1 determine discrete stages of neutrophil diapedesis. Immunity.

[12] Yang X., Zhao S., Wang S., Cao X., Xu Y., Yan M. (2023). Systemic inflammation indicators and risk of incident arrhythmias in 478,524 individuals: evidence from the UK Biobank cohort. BMC Med..

[13] Nøst T.H., Alcala K., Urbarova I., Byrne K.S., Guida F., Sandanger T.M. (2021). Systemic inflammation markers and cancer incidence in the UK Biobank. Eur. J. Epidemiol..

[14] Zhou Q., Liu J., Xin L., Hu Y., Qi Y. (2024). Systemic inflammation response index as an emerging biomarker in osteoarthritis patients: a bibliometric and large sample retrospective investigation. Clin. Exp. Rheumatol..

[15] Büyükavcı R., Aktürk S., Sağ S. (2018). Comparison of blood platelet distribution width and neutrophil-lymphocyte ratio in patients with different grades of knee osteoarthritis. J. Back Musculoskelet. Rehabil..

[16] Hsueh M.F., Zhang X., Wellman S.S., Bolognesi M.P., Kraus V.B. (2021). Synergistic roles of macrophages and neutrophils in osteoarthritis progression. Arthritis Rheumatol..

[17] Conroy M., Sellors J., Effingham M., Littlejohns T.J., Boultwood C., Gillions L. (2019). The advantages of UK Biobank's open-access strategy for health research. J. Intern. Med..

[18] Baker M.C., Weng Y., Robinson W.H., Ahuja N., Rohatgi N. (2020). Reduction in osteoarthritis risk after treatment with ticagrelor compared to clopidogrel: a propensity score-matching analysis. Arthritis Rheumatol..

[19] Terpstra S.E.S., van der Velde J., de Mutsert R., Schiphof D., Reijnierse M., Rosendaal F.R. (2021). The association of clinical and structural knee osteoarthritis with physical activity in the middle-aged population: the NEO study. Osteoarthritis Cartilage.

[20] Mutz J., Roscoe C.J., Lewis C.M. (2021). Exploring health in the UK Biobank: associations with sociodemographic characteristics, psychosocial factors, lifestyle and environmental exposures. BMC Med..

[21] Nedunchezhiyan U., Varughese I., Sun A.R., Wu X., Crawford R., Prasadam I. (2022). Obesity, inflammation, and immune system in osteoarthritis. Front. Immunol..

[22] Penninx B.W., Abbas H., Ambrosius W., Nicklas B.J., Davis C., Messier S.P. (2004). Inflammatory markers and physical function among older adults with knee osteoarthritis. J. Rheumatol..

[23] Jin X., Beguerie J.R., Zhang W., Blizzard L., Otahal P., Jones G. (2015). Circulating C reactive protein in osteoarthritis: a systematic review and meta-analysis. Ann. Rheum. Dis..

[24] Funck-Brentano T., Nethander M., Movérare-Skrtic S., Richette P., Ohlsson C. (2019). Causal factors for knee, hip, and hand osteoarthritis: a mendelian randomization study in the UK biobank. Arthritis Rheumatol..

[25] Bos S.D., Suchiman H.E., Kloppenburg M., Houwing-Duistermaat J.J., le Graverand M.P., Seymour A.B. (2008). Allelic variation at the C-reactive protein gene associates to both hand osteoarthritis severity and serum high sensitive C-reactive protein levels in the GARP study. Ann. Rheum. Dis..

[26] Kerkhof H.J., Bierma-Zeinstra S.M., Castano-Betancourt M.C., de Maat M.P., Hofman A., Pols H.A. (2010). Serum C reactive protein levels and genetic variation in the CRP gene are not associated with the prevalence, incidence or progression of osteoarthritis independent of body mass index. Ann. Rheum. Dis..

[27] Taşoğlu Ö., Bölük H., Şahin Onat Ş., Taşoğlu İ., Özgirgin N. (2016). Is blood neutrophil-lymphocyte ratio an independent predictor of knee osteoarthritis severity?. Clin. Rheumatol..

[28] Yombi J.C., Schwab P.E., Thienpont E. (2016). Neutrophil-to-lymphocyte ratio (NLR) distribution shows a better kinetic pattern than C-reactive protein distribution for the follow-up of early inflammation after total knee arthroplasty. Knee Surg. Sports Traumatol. Arthrosc..

[29] Valdrighi N., Vago J.P., Blom A.B., van de Loo F.A.J., Blaney Davidson E.N. (2022). Innate immunity at the core of sex differences in osteoarthritic pain?. Front. Pharmacol..

[30] Kalaitzoglou E., Griffin T.M., Humphrey M.B. (2017). Innate immune responses and osteoarthritis. Curr. Rheumatol. Rep..

[31] Wilkinson D.J., Falconer A.M.D., Wright H.L., Lin H., Yamamoto K., Cheung K. (2022). Matrix metalloproteinase-13 is fully activated by neutrophil elastase and inactivates its serpin inhibitor, alpha-1 antitrypsin: implications for osteoarthritis. FEBS J..

[32] Chaney S., Vergara R., Qiryaqoz Z., Suggs K., Akkouch A. (2022). The involvement of neutrophils in the pathophysiology and treatment of osteoarthritis. Biomedicines.

[33] Luo H., Zhu Y., Guo B., Ruan Z., Liu Z., Fan Z. (2023). Causal relationships between CD25 on immune cells and hip osteoarthritis. Front. Immunol..

[34] Xu J.P., Zeng R.X., Zhang Y.Z., Lin S.S., Tan J.W., Zhu H.Y. (2023). Systemic inflammation markers and the prevalence of hypertension: a NHANES cross-sectional study. Hypertens. Res..

[35] Zhang Y.R., Wang J.J., Chen S.F., Wang H.F., Li Y.Z., Ou Y.N. (2022). Peripheral immunity is associated with the risk of incident dementia. Mol. Psychiatr..

