# Supplementary material for: Association of peripheral inflammatory indicators with osteoarthritis risk
Source: Osteoarthr Cartil Open. 2024 Jun 19;6(3):100496. doi: 10.1016/j.ocarto.2024.100496 (PMC11254169; doi:10.1016/j.ocarto.2024.100496)
Supplement: Multimedia component 1 [file mmc1.docx]

**Supplementary materials**

**Association of Peripheral Inflammatory Indicators with Osteoarthritis Risk**

**Supplementary table 1**. ICD 10 codes for osteoarthritis in the UK Biobank.

| **ICD-10** | **Notes** |
| --- | --- |
| **Hand OA** |  |
| M15.1 | Heberden's nodes (with arthropathy) |
| M15.2 | Bouchard's nodes (with arthropathy) |
| M15.4 | Erosive (osteo)arthrosis |
| M18.0 | Primary arthrosis of first carpometacarpal joints, bilateral |
| M18.1 | Other primary arthrosis of first carpometacarpal joint |
| M18.9 | Arthrosis of first carpometacarpal joint, unspecified |
| M19.04 | Primary arthrosis of other joints (Hand) |
| M19.94 | Arthrosis, unspecified (Hand) |
| **Hip OA** |  |
| M16.0 | Primary coxarthrosis, bilateral |
| M16.1 | Other primary coxarthrosis |
| M16.9 | Coxarthrosis, unspecified |
| **Knee OA** |  |
| M17.0 | Primary gonarthrosis, bilateral |
| M17.1 | Other primary gonarthrosis |
| M17.9 | Gonarthrosis, unspecified |
| **Other OA** |  |
| M19.00 | Primary arthrosis of other joints (Multiple sites) |
| M19.01 | Primary arthrosis of other joints (Shoulder region) |
| M19.02 | Primary arthrosis of other joints (Upper arm) |
| M19.03 | Primary arthrosis of other joints (Forearm) |
| M19.05 | Primary arthrosis of other joints (Pelvic region and thigh) |
| M19.06 | Primary arthrosis of other joints-Lower leg |
| M19.07 | Primary arthrosis of other joints (Ankle and foot) |
| M19.08 | Primary arthrosis of other joints (Other) |
| M19.09 | Primary arthrosis of other joints (Site unspecified) |
| M19.90 | Arthrosis, unspecified (Multiple sites) |
| M19.91 | Arthrosis, unspecified (Shoulder region) |
| M19.92 | Arthrosis, unspecified (Upper arm) |
| M19.93 | Arthrosis, unspecified (Forearm) |
| M19.95 | Arthrosis, unspecified (Pelvic region and thigh) |
| M19.96 | Arthrosis, unspecified-Lower leg |
| M19.97 | Arthrosis, unspecified (Ankle and foot) |
| M19.98 | Arthrosis, unspecified (Other) |
| M19.99 | Arthrosis, unspecified (Site unspecified) |

**Supplementary table 2-1**. subgroups categorized by age.

|  | ≥60y | |  | <60y | |
| --- | --- | --- | --- | --- | --- |
|  | HR (95%CI) | *p* |  | HR (95%CI) | *p* |
| Neutrophils | 1.02 (1.01, 1.04) | <0.01 |  | 1.00 (0.98, 1.01) | 0.85 |
| lymphocytes | 0.96 (0.94, 0.98) | <0.01 |  | 0.99 (0.97, 1.02) | 0.51 |
| Monocytes | 1.01 (1.00, 1.03) | 0.07 |  | 1.00 (0.98, 1.02) | 0.99 |
| Platelets | 1.03 (1.01, 1.04) | <0.01 |  | 1.01 (1.00, 1.03) | 0.06 |
| C-reactive protein | 1.05 (1.04, 1.06) | <0.01 |  | 1.07 (1.06, 1.08) | <0.01 |
| NLR | 1.02 (1.02, 1.03) | <0.01 |  | 1.01 (0.99, 1.02) | 0.38 |
| PLR | 1.02 (1.01, 1.03) | <0.01 |  | 1.02 (1.00, 1.03) | 0.05 |
| SII | 1.03 (1.02, 1.04) | <0.01 |  | 1.01 (1.00, 1.03) | 0.15 |
| LMR | 0.96 (0.94, 0.98) | <0.01 |  | 0.98 (0.95, 1.00) | 0.04 |

**Supplementary table 2-2**. subgroups categorized by gender.

|  | Female | |  | Male | |
| --- | --- | --- | --- | --- | --- |
|  | HR (95%CI) | *p* |  | HR (95%CI) | *p* |
| Neutrophils | 1.02 (1.01, 1.04) | <0.01 |  | 1.00 (0.99, 1.02) | 0.69 |
| lymphocytes | 0.98 (0.96, 1.00) | 0.02 |  | 0.96 (0.94, 0.99) | <0.01 |
| Monocytes | 1.01 (1.00, 1.03) | 0.11 |  | 1.00 (0.98, 1.02) | 0.75 |
| Platelets | 1.03 (1.02, 1.04) | <0.01 |  | 1.02 (1.01, 1.04) | <0.01 |
| C-reactive protein | 1.07 (1.06, 1.08) | <0.01 |  | 1.04 (1.03, 1.05) | <0.01 |
| NLR | 1.03 (1.02, 1.04) | <0.01 |  | 1.01 (1.00, 1.02) | 0.09 |
| PLR | 1.03 (1.02, 1.04) | <0.01 |  | 1.01 (1.00, 1.02) | <0.01 |
| SII | 1.04 (1.03, 1.05) | <0.01 |  | 1.01 (1.00, 1.03) | <0.01 |
| LMR | 0.97 (0.95, 0.98) | <0.01 |  | 0.98 (0.95, 1.00) | 0.05 |

**Supplementary table 2-3**. subgroups categorized by obesity.

|  | Normal | |  | Obesity | |
| --- | --- | --- | --- | --- | --- |
|  | HR (95%CI) | *p* |  | HR (95%CI) | *p* |
| Neutrophils | 1.00 (0.99, 1.02) | 0.62 |  | 1.02 (1.01, 1.04) | <0.01 |
| lymphocytes | 0.97 (0.95, 0.99) | <0.01 |  | 0.97 (0.95, 0.99) | <0.01 |
| Monocytes | 1.01 (0.99, 1.03) | 0.36 |  | 1.00 (0.99, 1.02) | 0.70 |
| Platelets | 1.03 (1.02, 1.04) | <0.01 |  | 1.02 (1.00, 1.03) | 0.01 |
| C-reactive protein | 1.05 (1.04, 1.06) | <0.01 |  | 1.06 (1.05, 1.07) | <0.01 |
| NLR | 1.02 (1.00, 1.03) | <0.01 |  | 1.02 (1.02, 1.03) | <0.01 |
| PLR | 1.03 (1.02, 1.04) | <0.01 |  | 1.02 (1.01, 1.03) | <0.01 |
| SII | 1.02 (1.01, 1.04) | <0.01 |  | 1.02 (1.01, 1.03) | <0.01 |
| LMR | 0.97 (0.95, 0.99) | <0.01 |  | 0.97 (0.95, 0.99) | <0.01 |

*According to the World Health Organization definition, a BMI ≥ 29.9 kg/m^2^ is considered obesity.

**Supplementary table 2-4**. subgroups categorized by smoking.

|  | Non-smoker | |  | Smoker | |
| --- | --- | --- | --- | --- | --- |
|  | HR (95%CI) | *p* |  | HR (95%CI) | *p* |
| Neutrophils | 1.01 (0.99, 1.02) | 0.37 |  | 1.01 (1.00, 1.02) | 0.14 |
| lymphocytes | 0.97 (0.95, 0.99) | <0.01 |  | 0.96 (0.94, 0.98) | <0.01 |
| Monocytes | 1.01 (0.99, 1.02) | 0.36 |  | 1.00 (0.98, 1.02) | 0.14 |
| Platelets | 1.03 (1.01, 1.04) | <0.01 |  | 1.03 (1.01, 1.04) | <0.01 |
| C-reactive protein | 1.06 (1.05, 1.07) | <0.01 |  | 1.05 (1.04, 1.07) | <0.01 |
| NLR | 1.02 (1.01, 1.03) | <0.01 |  | 1.02 (1.01, 1.03) | <0.01 |
| PLR | 1.02 (1.01, 1.03) | <0.01 |  | 1.03 (1.02, 1.04) | <0.01 |
| SII | 1.02 (1.01, 1.03) | <0.01 |  | 1.03 (1.02, 1.04) | <0.01 |
| LMR | 0.96 (0.94, 0.98) | <0.01 |  | 0.97 (0.95, 0.99) | 0.01 |

*Individuals with a current or previous smoking status were defined as smokers.

**Supplementary table 2-5**. subgroups categorized by drinking.

|  | Non-drinker | |  | Drinker | |
| --- | --- | --- | --- | --- | --- |
|  | HR (95%CI) | *p* |  | HR (95%CI) | *p* |
| Neutrophils | 1.03 (1.01, 1.05) | <0.01 |  | 1.01 (1.00, 1.02) | 0.16 |
| lymphocytes | 0.98 (0.95, 1.01) | 0.15 |  | 0.97 (0.95, 0.98) | <0.01 |
| Monocytes | 1.00 (0.98, 1.03) | 0.79 |  | 1.01 (0.99, 1.02) | 0.41 |
| Platelets | 1.04 (1.02, 1.06) | <0.01 |  | 1.02 (1.01, 1.03) | <0.01 |
| C-reactive protein | 1.05 (1.04, 1.07) | <0.01 |  | 1.06 (1.05, 1.07) | <0.01 |
| NLR | 1.03 (1.01, 1.04) | <0.01 |  | 1.02 (1.01, 1.02) | <0.01 |
| PLR | 1.03 (1.02, 1.04) | <0.01 |  | 1.02 (1.01, 1.03) | <0.01 |
| SII | 1.04 (1.02, 1.05) | <0.01 |  | 1.02 (1.01, 1.04) | <0.01 |
| LMR | 0.97 (0.95, 1.00) | 0.05 |  | 0.97 (0.95, 0.98) | 0.01 |

*Individuals who rarely or never drink were defined as non-drinkers.

**Supplementary table 2-6**. subgroups categorized by activity level.

|  | Low level | |  | High level | |
| --- | --- | --- | --- | --- | --- |
|  | HR (95%CI) | *p* |  | HR (95%CI) | *p* |
| Neutrophils | 1.02 (1.01, 1.04) | <0.01 |  | 1.01 (1.00, 1.02) | 0.03 |
| lymphocytes | 0.98 (0.96, 1.01) | 0.21 |  | 0.97 (0.95, 0.98) | <0.01 |
| Monocytes | 1.01 (0.99, 1.03) | 0.35 |  | 1.00 (0.99, 1.02) | 0.57 |
| Platelets | 1.03 (1.01, 1.05) | <0.01 |  | 1.03 (1.02, 1.04) | <0.01 |
| C-reactive protein | 1.07 (1.06, 1.08) | <0.01 |  | 1.05 (1.04, 1.06) | <0.01 |
| NLR | 1.02 (1.00, 1.03) | <0.01 |  | 1.02 (1.01, 1.03) | <0.01 |
| PLR | 1.02 (1.01, 1.03) | <0.01 |  | 1.02 (1.02, 1.03) | <0.01 |
| SII | 1.02 (1.01, 1.03) | <0.01 |  | 1.03 (1.02, 1.04) | <0.01 |
| LMR | 0.97 (0.94, 0.99) | 0.02 |  | 0.97 (0.95, 0.99) | <0.01 |

*Activity levels were categorized according to Metabolic Equivalent of Task (MET), with those engaging in 600 minutes or more per week defined as having high levels of exercise.

**Supplementary table 2-7**. subgroups categorized by income level.

|  | Low level | |  | High level | |
| --- | --- | --- | --- | --- | --- |
|  | HR (95%CI) | *p* |  | HR (95%CI) | *p* |
| Neutrophils | 0.99 (0.98, 1.01) | 0.54 |  | 1.03 (1.01, 1.04) | <0.01 |
| lymphocytes | 0.95 (0.92, 0.97) | <0.01 |  | 0.98 (0.96, 1.00) | 0.21 |
| Monocytes | 1.00 (0.98, 1.02) | 0.83 |  | 1.01 (1.00, 1.02) | 0.03 |
| Platelets | 1.01 (1.00, 1.03) | 0.09 |  | 1.03 (1.02, 1.05) | <0.01 |
| C-reactive protein | 1.06 (1.05, 1.08) | <0.01 |  | 1.06 (1.05, 1.07) | <0.01 |
| NLR | 1.02 (1.01, 1.04) | <0.01 |  | 1.02 (1.01, 1.03) | <0.01 |
| PLR | 1.04 (1.02, 1.05) | <0.01 |  | 1.02 (1.01, 1.03) | <0.01 |
| SII | 1.03 (1.01, 1.04) | <0.01 |  | 1.03 (1.02, 1.03) | <0.01 |
| LMR | 0.97 (0.94, 1.00) | 0.03 |  | 0.97 (0.95, 0.99) | <0.01 |

*Individuals with an annual income greater than £30,999 were defined as having a high income level.

**Supplementary table 2-8**. subgroups categorized by education level.

|  | Low level | |  | High level | |
| --- | --- | --- | --- | --- | --- |
|  | HR (95%CI) | *p* |  | HR (95%CI) | *p* |
| Neutrophils | 1.02 (1.01, 1.03) | <0.01 |  | 1.01 (1.00, 1.03) | 0.14 |
| lymphocytes | 0.98 (0.96, 1.00) | 0.01 |  | 0.96 (0.94, 0.99) | <0.01 |
| Monocytes | 1.00 (0.99, 1.02) | 0.54 |  | 1.01 (0.99, 1.03) | 0.03 |
| Platelets | 1.03 (1.02, 1.04) | <0.01 |  | 1.03 (1.02, 1.05) | 0.14 |
| C-reactive protein | 1.06 (1.05, 1.07) | <0.01 |  | 1.06 (1.05, 1.08) | <0.01 |
| NLR | 1.02 (1.01, 1.02) | <0.01 |  | 1.03 (1.01, 1.05) | <0.01 |
| PLR | 1.02 (1.01, 1.03) | <0.01 |  | 1.03 (1.01, 1.04) | <0.01 |
| SII | 1.02 (1.02, 1.03) | <0.01 |  | 1.03 (1.01, 1.05) | <0.01 |
| LMR | 0.98 (0.96, 0.99) | <0.01 |  | 0.96 (0.93, 0.99) | <0.01 |

*Individuals with a college or university degree, or other professional qualifications, were defined as having a higher education level.

**Supplementary table 3**. Association between peripheral inflammatory markers and risks of specific joint OA.

|  | **Hand OA** | |  | **Hip OA** | |  | **Knee OA** | |
| --- | --- | --- | --- | --- | --- | --- | --- | --- |
|  | HR (95%CI) | *p* |  | HR (95%CI) | *p* |  | HR (95%CI) | *p* |
| Neutrophils | 1.03 (1.00, 1.07) | 0.09 |  | 1.01 (1.00, 1.03) | 0.10 |  | 0.99 (0.98, 1.01) | 0.23 |
| lymphocytes | 1.00 (0.95, 1.05) | 0.89 |  | 0.91 (0.88, 0.94) | < 0.01 |  | 0.99 (0.97, 1.01) | 0.16 |
| Monocytes | 1.02 (0.98, 1.06) | 0.31 |  | 0.99 (0.97, 1.01) | 0.39 |  | 1.00 (0.99, 1.02) | 0.72 |
| Platelets | 1.06 (1.02, 1.09) | < 0.01 |  | 1.02 (1.00, 1.03) | 0.05 |  | 1.02 (1.00, 1.03) | 0.02 |
| C-reactive protein | 1.03 (1.00, 1.06) | 0.06 |  | 1.04 (1.02, 1.05) | < 0.01 |  | 1.06 (1.05, 1.07) | < 0.01 |
| NLR | 1.02 (1.00, 1.05) | 0.04 |  | 1.03 (1.02, 1.04) | < 0.01 |  | 1.00 (0.99, 1.02) | 0.72 |
| PLR | 1.03 (1.00, 1.05) | 0.01 |  | 1.03 (1.02, 1.04) | < 0.01 |  | 1.01 (1.00, 1.02) | 0.02 |
| SII | 1.04 (1.01, 1.06) | < 0.01 |  | 1.03 (1.02, 1.04) | < 0.01 |  | 1.01 (1.00, 1.02) | 0.05 |
| LMR | 1.00 (0.96, 1.04) | 0.85 |  | 0.93 (0.90, 0.96) | < 0.01 |  | 0.98 (0.96, 1.00) | 0.03 |

**Supplementary table 4. Risk of osteoarthritis according to peripheral inflammatory indicators after propensity score matching.**

|  | **Low** | **High** |  |
| --- | --- | --- | --- |
|  |  | **Hazard Ratio (95% CI)** | ***p*** |
| Neutrophils | Reference | 1.10 (1.08, 1.12) | < 0.01 |
| lymphocytes | Reference | 0.98 (0.96, 0.99) | < 0.01 |
| Monocytes | Reference | 1.05 (1.03, 1.07) | < 0.01 |
| Platelets | Reference | 1.10 (1.07, 1.14) | < 0.01 |
| C-reactive protein | Reference | 1.31 (1.28, 1.33) | < 0.01 |
| NLR | Reference | 1.04 (1.02, 1.06) | < 0.01 |
| PLR | Reference | 1.01 (1.00, 1.03) | 0.03 |
| SII | Reference | 1.07 (1.05, 1.09) | < 0.01 |
| LMR | Reference | 0.98 (0.97, 0.99) | 0.01 |

**Supplementary table 5**. Excluded participants with less than 2 and 5 years of follow-up respectively.

|  | ≥2 years | |  | ≥5 years | |
| --- | --- | --- | --- | --- | --- |
|  | HR (95%CI) | *p* |  | HR (95%CI) | *p* |
| Neutrophils | 1.01 (1.00,1.02) | 0.02 |  | 1.01 (1.00,1.02) | 0.06 |
| lymphocytes | 0.97 (0.95,0.99) | <0.01 |  | 0.97 (0.96,0.99) | <0.01 |
| Monocytes | 1.01 (0.99,1.02) | 0.33 |  | 1.00 (0.99,1.02) | 0.72 |
| Platelets | 1.03 (1.02,1.04) | <0.01 |  | 1.02 (1.01,1.04) | <0.01 |
| C-reactive protein | 1.05 (1.05,1.06) | <0.01 |  | 1.05 (1.04,1.06) | <0.01 |
| NLR | 1.02 (1.01,1.03) | <0.01 |  | 1.02 (1.01,1.03) | <0.01 |
| PLR | 1.02 (1.01,1.03) | <0.01 |  | 1.02 (1.01,1.03) | <0.01 |
| SII | 1.02 (1.02,1.03) | <0.01 |  | 1.02 (1.01,1.04) | <0.01 |
| LMR | 0.97 (0.95,0.98) | <0.01 |  | 0.97 (0.96,0.99) | <0.01 |


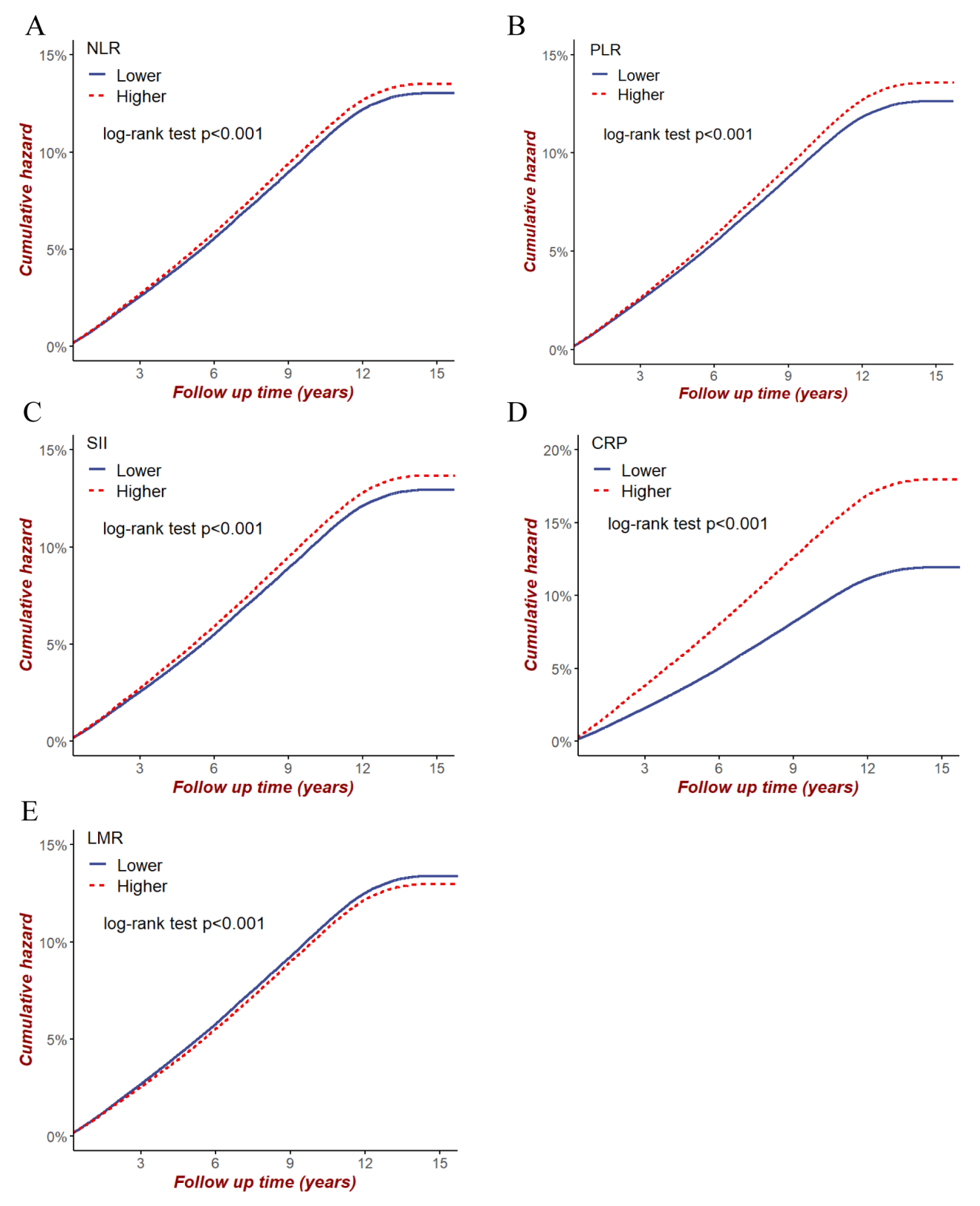


**Supplementary figure 1**. Kaplan-Meier survival analysis of cumulative risk of OA according to NLR (A), PLR (B), SII (C), CRP (D), and LMR (E) status. Cutoff values for the lower and higher groups were defined as the median of the whole group.
